# Determination of Paclitaxel Distribution in Solid Tumors by Nano-Particle Assisted Laser Desorption Ionization Mass Spectrometry Imaging

**DOI:** 10.1371/journal.pone.0072532

**Published:** 2013-08-26

**Authors:** Lavinia Morosi, Pietro Spinelli, Massimo Zucchetti, Francesca Pretto, Andrea Carrà, Maurizio D’Incalci, Raffaella Giavazzi, Enrico Davoli

**Affiliations:** 1 IRCCS Istituto di Ricerche Farmacologiche “Mario Negri”, Department of Oncology, Milano, Italy; 2 IRCCS Istituto di Ricerche Farmacologiche “Mario Negri”, Department of Environmental Health Sciences, Mass Spectrometry Laboratory, Milano, Italy; Virginia Commonwealth University, United States of America

## Abstract

A sensitive, simple and reproducible protocol for nanoparticle-assisted laser desorption/ionization mass spectrometry imaging technique is described. The use of commercially available TiO_2_ nanoparticles abolishes heterogeneous crystallization, matrix background interferences and enhances signal detection, especially in the low mass range. Molecular image normalization was based on internal standard deposition on tissues, allowing direct comparison of drug penetration and distribution between different organs and tissues. The method was applied to analyze the distribution of the anticancer drug paclitaxel, inside normal and neoplastic mouse tissue sections. Spatial resolution was good, with a linear response between different *in vivo* treatments and molecular imaging intensity using therapeutic drug doses. This technique distinguishes the different intensity of paclitaxel distribution in control organs of mice, such as liver and kidney, in relation to the dose. Animals treated with 30 mg/kg of paclitaxel had half of the concentration of those treated with 60 mg/kg. We investigated the spatial distribution of paclitaxel in human melanoma mouse xenografts, following different dosage schedules and found a more homogeneous drug distribution in tumors of mice given repeated doses (5×8 mg/kg) plus a 60 mg/kg dose than in those assigned only a single 60 mg/kg dose. The protocol can be readily applied to investigate anticancer drug distribution in neoplastic lesions and to develop strategies to optimize and enhance drug penetration through different tumor tissues.

## Introduction

Mass spectrometry imaging (MSI) is one of the latest, rapidly growing, innovative techniques in mass spectrometry (MS) [1]. It is used to visualize molecular distribution in a two-dimensional space of a sample attached to an electric conductive steel plate or glass slide. The impressive growth of publications [2] reflects the success of this technique in different fields. In the biomedical sciences it is used in applied biology, pathology, medicine and pharmacology. Images can be obtained from single cells to whole animal sections, from plants, bacteria, animals and human tissues [3], [4], [5], [6], [7]. In a typical MSI experiment, a tissue section is deposited on a steel plate, sprayed with a matrix solution and analyzed by matrix-assisted laser desorption/ionization time-of-flight mass spectrometer (MALDI-TOF). The distribution of biomolecules on a tissue section can be readily visualized in two-dimensions, assigning to each pixel the ion intensity specific for the molecule under study. As each individual image pixel can be a full mass spectrum, a number of images can be created from a single experiment, just selecting the different mass values available in the spectrum.

MSI has rapidly emerged as an appealing and valuable technology for the localization of drugs and small molecules in biological tissue [8]. The unique advantage of MSI for drugs, compared to standard detection methods, is that the spatial distribution of an unlabeled drug and its metabolites can be studied without any preparation step on the tissue under analysis. A limited number of MSI studies have shown the distribution of some drugs in animal tissues [9], [10], [11], [12], [13] or human biopsies [14].

Two drawbacks slowed the development of MSI in this field: the high background noise in the low mass region, due to the standard MALDI matrixes commonly used and the high abundance of endogenous molecules with similar molecular weights, that contribute to masking the analytes’ ion signal [2].

The use of nanoparticles (NPs) as matrixes in MALDI imaging has opened up new opportunities thanks to the almost complete absence of background signals from matrix degradation. The use of inorganic fine particles [15] is based on their physical properties, like high photo-adsorption, low heat capacity and large surface area. This ensures rapid heating [16], highly localized and uniform energy deposition [17], resulting in efficient sample desorption and ionization. Moreover, nanoparticle-assisted laser desorption ionization (nPALDI), when used for MSI, provides better spatial resolution than conventional matrixes. Here the crystallization is eliminated and the maximum resolution is not limited by crystal size but only by instrumental specifications, like laser spot diameter, for example [18]. Previously our group was able to obtain images with spatial resolution up to 20 µm [19]. Metallic NPs have been used to image several tissues [20], [21], [22], [23], [24], and TiO_2_ has been employed to study the distribution of small endogenous molecules inside human breast cancer xenografts in mice and mouse brain [18], [25].

Drug imaging is very important in oncology where the distribution within tumor tissue is thought to play a pivotal role in response to therapy and could partly explain the variable response rates, often even in different patients with similar tumor types [26]. Concentrations of anticancer drugs in tumor are measured by homogenization-based techniques that, although sensitive, do not give spatial information and do not understand the drug distribution in the specific organization of tumor tissue. Despite the potential sensitivity and spatial resolution attainable in tumor samples [27], only a few MSI studies have looked at anti-cancer agent distribution in tumor samples [28], [29].

We have developed a simple nPALDI MSI protocol, using commercially available TiO2 NPs, for the qualitative and quantitative analysis of paclitaxel (PTX), and to study its distribution in tumor tissues. PTX is an alkaloid that promotes microtubule assembly and inhibits microtubule disassembly, inducing mitotic arrest [30]. It is commonly used as a anticancer agent, given every 3–4 weeks, for the treatment of different solid tumors, mainly ovarian and breast cancer [31], [32]. It has been recently documented that a different schedule based on dose-dense weekly paclitaxel gives better response and survival [33] and the patients benefit from the chronic exposure to taxanes before receiving high-dose treatment with other drugs to maximize tumor response [34], [35].

The aim of this study was to develop a protocol that allowed us the imaging of PTX after i.v. administration, in normal organs or tumor tissue of mice, with high spatial resolution and sensitivity. With this method we have observed differences in the distribution of PTX in tumors, related to the dosage-schedule.

## Materials and Methods

### Cell Lines, Drugs and Reagents

WM1552/5 is a tumorigenic variant derived from WM1552 human melanoma [36], and 1A9 is a variant derived from A2780 human ovarian carcinoma cell line [37]. We confirmed the identity of the variants with the parental cell line by DNA fingerprinting analysis (short tandem repeat profiling, AmpFISTR Identifiler Plus PCR Amplification Kit, Applied Biosystems). Stocks of cell lines were stored frozen in liquid nitrogen and kept in culture for no more than eight weeks before injection.

Tumor cells were cultured in RPMI 1640 (Gibco, Paisley, UK) supplemented with 10% heat-inactivated fetal calf serum (Sigma, St. Louis, MO, USA) and 1% L-glutamine (Gibco) and maintained in a humidified atmosphere with 5% CO_2_ at 37°C. Exponentially growing cells were harvested, repeatedly washed and re-suspended in serum-free medium before injection.

Paclitaxel (PTX, Indena S.p.A., Milan, Italy) and paclitaxel-D5 (D5-PTX, Toronto Research, Canada) were dissolved in 50% ethanol at a concentration of 0.1 to 100 pmol/µL for all MS experiments; for mouse treatment PTX was dissolved in 50% Cremophor EL (Sigma) and 50% ethanol and further diluted in saline immediately before use. PTX was administered intravenously (i.v.) at a dose of 30–60 mg/kg or as five daily dose of 8 mg/kg.

TiO_2_ nanoparticles (Aeroxide® TiO_2_ P 25, Evonik Industrials, Essen, Germany) were used as a matrix for MSI experiments. For validation tests, a suspension was prepared at 1 mg/mL in MilliQ water; for MSI a solution of 1 mg/mL in ethanol 50%/NaCl 0.9% was used. TiO_2_ nanoparticles have very high agglomeration rate in water and it depends closely on the physicochemical water parameters [38]. To ensure a reproducible aqueous suspension, the solution was vortexed and sonicated for 3 min just before use, to reduce agglomeration and sedimentation. Saline solution (NaCl 0.9%) was used to prevent cell lysis and tissue degradation when the matrix was applied and ethanol was used to facilitate and speed up solvent evaporation during matrix deposition.

### Mice and Human Xenografts

Procedures involving animals and their care were conducted in conformity with the institutional guidelines that are in compliance with national (Legislative Decree 116 of Jan. 27, 1992 Authorisation n.169/94-A issued Dec. 19, 1994 by Ministry of Health) and international laws and policies (EEC Council Directive 86/609, OJ L 358. 1, December 12, 1987; Standards for the Care and Use of Laboratory Animals, United States National Research Council, Statement of Compliance A5023-01, November 6, 1998). Animal experiments has been reviewed and approved by the Istituto di Ricerche Farmacologiche Mario Negri Animal Care and Use Committee (IACUC) that includes members “ad hoc” for ethical issues. Animals were housed in the Institute’s Animal Care Facilities, which meet international standards; they are regularly checked by a certified veterinarian who is responsible for health monitoring, animal welfare supervision, experimental protocols and procedures revision. Animal were sacrificed under CO_2_, and all efforts were made to minimize suffering.

Six- to eight-week-old female NCr-nu/nu mice (Harlan, Correzzana, Italy) were used. To validate the tissue imaging protocol in organs (i.e. liver, kidney), mice were treated with vehicle (control, CTRL) or with PTX (30 or 60 mg/kg i.v.) and sacrificed after 15 minutes. Livers and kidneys were frozen in liquid nitrogen and stored at −80°C until MSI.

For melanoma and ovarian carcinoma xenografts, WM1552/5 (2×10^6^ tumor cells) or A2780-1A9 (10×10^6^ tumor cells) cells were injected subcutaneously in the flank of nude mice. Mice bearing melanoma xenografts (approximately 750 mg) were treated either with vehicle (CTRL), with five daily doses (8 mg/Kg, i.v) followed by a final dose (60 mg/kg i.v.) of PTX, or with a single dose of PTX (60 mg/kg i.v.) and sacrificed 1 h after the last treatment. Mice bearing ovarian carcinoma xenografts (approximately 750 mg) were treated with vehicle (CTRL) or with PTX (60 mg/kg i.v.) and sacrificed 6 h after treatment. Tumors were explanted then immediately snap-frozen in liquid nitrogen and stored at −80°C until further analysis.

### Sample Preparation for MSI

Frozen tissues were cut in 14 µm thick sections using a cryo-microtome (Leica Microsystems, Wetzler, Germany ) at −20°C. Three to five sections were cut from the central part of every tumor or organ and were mounted on a –20°C pre-cooled MALDI plate (Opti-TOF 384 Well insert) by standard thaw-mounting techniques, gently touching the back of the frozen MALDI plate with a finger and stored at −80°C until further analysis. For each section, two adjacent sections were cut, one to be imaged in MS/MS mode and the other (10 µm thickness) placed on a glass slide for hematoxylin and eosin (HE) staining. The plate with the tissue sections was dried in a vacuum drier at room temperature for 1 h. Different control spots of PTX and D5-PTX were applied on the tissue sections, to be used as reference for instrumental calibration or quantification (see “Quantitative analysis by MSI”). The plate was scanned in high optical resolution to obtain images of the sections, then sprayed with TiO_2_ matrix suspension using a BD 180 precision double-action trigger airbrush (Fenga, Mexico) with a 0.20 mm nozzle diameter, using nitrogen at 0.2 atm. Care was taken to avoid over-spraying the matrix suspension on a single point so as to avoid the formation of droplets that would wet the surface with possible damage of the tissue structure, analyte diffusion or (partial) detachment of the slice from the plate.

### MS, MS/MS and MSI Analysis of PTX by n-PALDI

A MALDI 4800 TOF-TOF (AB SCIEX Old Connecticut Path, Framingham, MA 01701, USA) was used, equipped with a 355 nm Nd:YAG laser with a 200 Hz repetition rate, controlled by the 4000 Series Explorer™ software (AB SCIEX Old Connecticut Path, Framingham, MA 01701, USA). MS and MS/MS spectra were acquired with 20 laser shots with intensity of 6000 arbitrary units, with a bin size of 0.5–1.0 ns, acquiring in reflectron, both negative and positive-ion mode. The instrument was mass calibrated with a CAL-MIX before analysis and used at 10,000 resolving power (RP).

The initial method setup for nPALDI of PTX standard simply involved depositing 1 µL of PTX or D5-PTX 1 pmol/µL on the steel MALDI plate (Opti-TOF 384 Well insert) waiting until it was dried, followed by 1 µL of TiO_2_ matrix suspension on top. Images of tissue sections were acquired using the 4800 Imaging Tool software (www.maldi-msi.org, M. Stoeckli, Novartis Pharma, Basel, Switzerland), spectra were acquired with an imaging raster of 75×75 microns, on at least 3 sections per tissue or organ.

**Figure 1:**
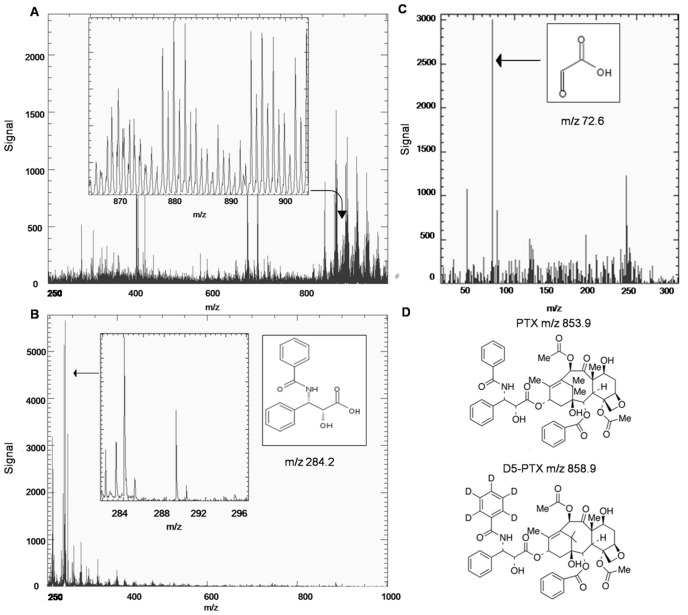
MS and MS/MS spectra of PTX. MALDI mass spectrum of a PTX spot (10 pmol) on tumor tissue sections in positive ion mode (**A**) and negative ion mode (**B**). In positive ion mode a lot of background signals in the m/z 800–1000 mass range cover the paclitaxel peak (m/z 877 or 892). In negative ion mode, however, ion m/z 284.2 is clearly predominant in the spectrum (inset zoomed view). (**C**) MS/MS spectrum from fragmentation of ion m/z 284.2, glyoxylic anion at m/z 72.6 is shown (arrow). (**D**) Chemical structures of PTX and D5-PTX.

TissueView software 1.1 (AB SCIEX Old Connecticut Path, Framingham, MA 01701, USA) was used to process and display ions distribution inside the tumor sections. In MS experiments PTX and D5-PTX were imaged in negative mode by plotting fragment ions at m/z 284.2 and m/z 289.2 respectively, corresponding to the side chain with the amide-acyl group (Figure 1). PTX was also imaged in MS/MS, mode monitoring the transition m/z 284.2 to m/z 72.6. Fragmentation patterns were verified with a continuum infusion on LTQ Orbitrap high resolution MS (Thermo Fisher Scientific Inc, USA) equipped with an electrospray ion source (OmniSpray, Prosolia Inc., USA) operated at 10,000 RP. The molecular negative ion has been fragmented by collision-induced dissociation (CID) to produce MS^2^ and MS^3^ spectra to confirm product ions.

### Quantitative Analysis by MSI

To quantify PTX in tissue sections we plotted a calibration curve. Different amounts of PTX standard solution (1–15 pmol) and a fixed concentration of internal standard D5-PTX (5 pmol) were applied by carefully spotting 0.2 µL on control dried tissues, mounted on the MALDI plate, to a final dry spot of about 2 mm^2^. Average intensity was calculated for PTX and the internal standard D5-PTX, drawing a region of interest (ROI) around each spot. A concentration per surface unit in pmol/mm^2^ was obtained by dividing the spotted quantity by the area of the corresponding ROI. The mean PTX/D5-PTX ion intensity ratio was plotted against the spotted PTX amount per surface.

To normalize PTX signals in differently treated tissue sections, we adjust the color scale maximizing the mean intensity of the internal standard spotted on tissue (highest scale bar color). Knowing that the internal standard spot corresponds to 5 pmol/mm^2^ as the standard spot area was constant in the different sections (mean area was 1.2+/−0.15 mm^2^), PTX visualization was standardized to a 0–5 pmol/mm^2^ scale for all different tissue sections, taking account of the possible differences in absolute intensities, due to instrumental variations, but also from differences in tissue responses [39].

### Absolute Quantification and Validation of Drug Identification

A conventional quantitative approach was used to measure drug residues in tumor samples of differently treated animals. Adjacent melanoma sections were weighed and homogenized in 50% aqueous acetonitrile containing internal standard, then vortexed and centrifuged. The supernatant was collected and diluted with acetonitrile. HPLC-MS/MS analysis was done as previously described [40]. The instrument available was an API 3000 (AB SCIEX Old Connecticut Path, Framingham, MA 01701, USA) triple quadrupole mass spectrometer equipped with a Perkin Elmer Series 200 HPLC. A Waters XTerra MS C18 column, 100×2.1 mm i.d. was used for chromatographic separation. The mobile phases were 0.01% formic acid in MilliQ water (A) and acetonitrile (B), with a gradient from 40% A to 66% B in 7 min at 200 µL/min. The Turbo Ion spray source temperature was 400°C, the IS voltage was −4500 V. A declustering potential of −80 V and a focusing potential of −200 V were used. Mass spectrometry analyses were done in Multiple Reaction Monitoring (MRM) mode. PTX and D5-PTX eluted at 6.4 min with no interfering peaks. Transitions monitored were m/z 898 [M-H+HCOOH]^−^ to 525 for PTX (corresponding to the loss of CH_3_COOH from the pentadecene ring system) and m/z 903 [M-H+HCOOH]^−^ to 525 for D5-PTX, with a collision energy of 25 eV.

## Results and Discussion

### Identification of PTX Residues with MALDI TOF/TOF using TiO_2_ Nanoparticles

In MALDI experiments PTX is observed in positive ion mode as an adduct with Na+ or K+ at m/z 877 and 892 respectively [41], [42]. With TiO_2_ nanoparticles cationization readily occurs, as it is the predominant ionization mechanism for several compounds [43] **.** In that mass region, tissue imaging showed limited sensitivity, precluding successful PTX MSI because of the high background noise, mainly due to lipids (Figure 1A). Using nPALDI and negative ions, PTX efficiently ionizes and fragments in the ion source, to produce ions at m/z 284.2 (289.2 for the internal standard D5-PTX) as base peaks, corresponding to the side chain fragment, after the loss of the pentadecene ring system (Figure 1B). Under MS/MS CID the side chain dissociates to produce the glyoxylic anion at m/z 72.6 (Figure 1C). We validated the ion elemental composition for these fragments by direct infusion high-resolution MS^2^ and MS^3^ experiments with the correct atomic composition assigned with <5 ppm error for the mass m/z 284.0926 and with 109 ppm error for the mass m/z 73.0000 (Figure S1, S2 and S3 in File S1).

### Calibration Curve and Quantification in Normal Tissues using MSI

**Figure 2:**
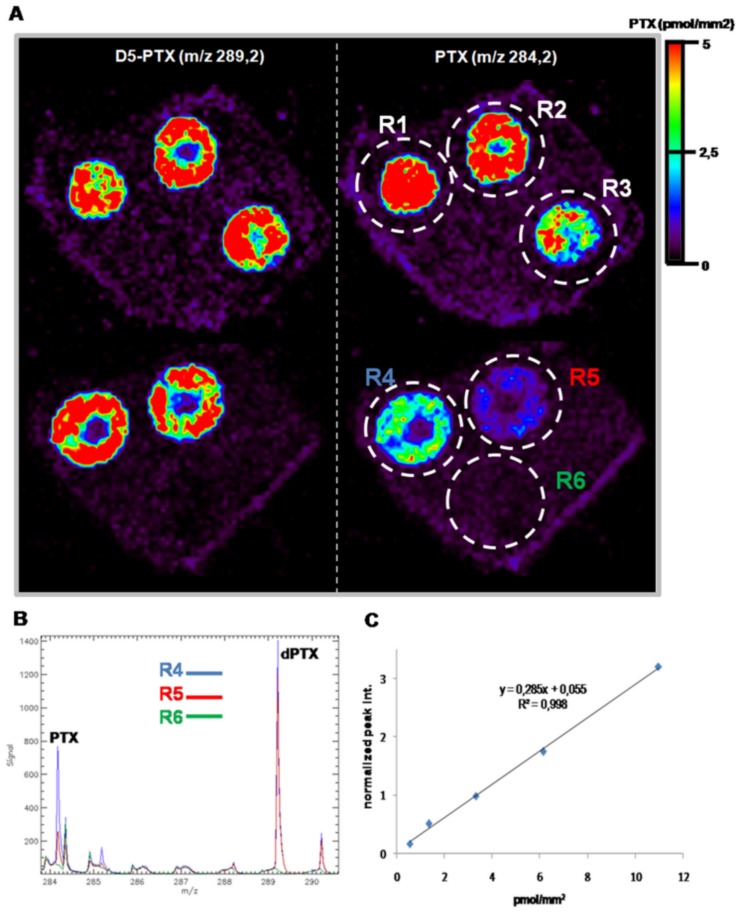
Calibration curve. **A)** Increasing amounts of PTX (1-2.5-5-10-15 pmol) were co-spotted with a constant amount of D5-PTX (5 pmol) on control liver sections. Ion images of PTX (ion m/z 284.2, right) and D5-PTX (ion 289 m/z, left) are shown. **B**) Six region of interests (ROIs) were drawn around the spots and the mean spectra of three ROIs are shown. PTX peak at m/z 284.2 decrease from R4 to R6 while the peak at 289.2 remains almost constant. R6 is the background signal. **C)** Standard curve plotted using the mean signal intensity ratio (PTX/D5-PTX).

We verified the sensitivity and linearity of the response by spotting increasing concentrations of PTX (1–15 pmol), on liver and kidney slices of control mice, with a fixed amount of D5-PTX (5 pmol), and imaging the side chain distribution at m/z 284.2 for the drug and 289.2 for its internal standard. Images were created by plotting ions in the region of +/−0.1 amu in order to gain specificity (Figure 2A). At similar nominal masses other ions are present that could interfere with PTX signal, limiting the specificity and, therefore, image quality. For all tissues the limit of detection (LOD) was 1 pmol/spot, equivalent to approximately 0.5 pmol/mm^2^. Different ROIs used to average ion intensities for each spot are marked R1–R6, the last being the background ROI used for background subtraction for PTX and D5-PTX signal (Figure 2B). Figure 2C shows a calibration curve built on a control liver sample. The inter-day reproducibility of the calibration curve was verified repeating the experiment on a more complex biological matrix as tumor tissue (ovarian carcinoma xenografts A2780-1A9). No significant variability was detected in three different days (Figure S4 in File S1). Relative standard deviation percentage (±RSD%) was calculated to assess sample-to-sample reproducibility in terms of precision of the method. The concentration of 5 pmol/spot of PTX and D5-PTX was spotted on three serial section of tumor tissue and imaging experiment was performed. The mean PTX/D5-PTX ion intensity ratio of the triplicated spot was used to calculate RSD% (SD/mean *100) that resulted 9.37%, a small variation in signal intensity [18].

We tested the protocol by analyzing PTX distribution inside kidney and liver, as representative tissues of mice treated with the anticancer drug at 30 or 60 mg/kg i.v. and sacrificed after 15 minutes. The use of an internal standard on-tissue spotting approach allows concentration normalization between different slices and different tissues. It is worth to note that where internal standard is spotted PTX, signal is slightly suppressed, possibly due to competing processes in the ionization mechanism. Drug penetration and distribution can be directly compared in tissues even when there are different ion suppression effects in a particular organ or region of interest.

**Figure 3:**
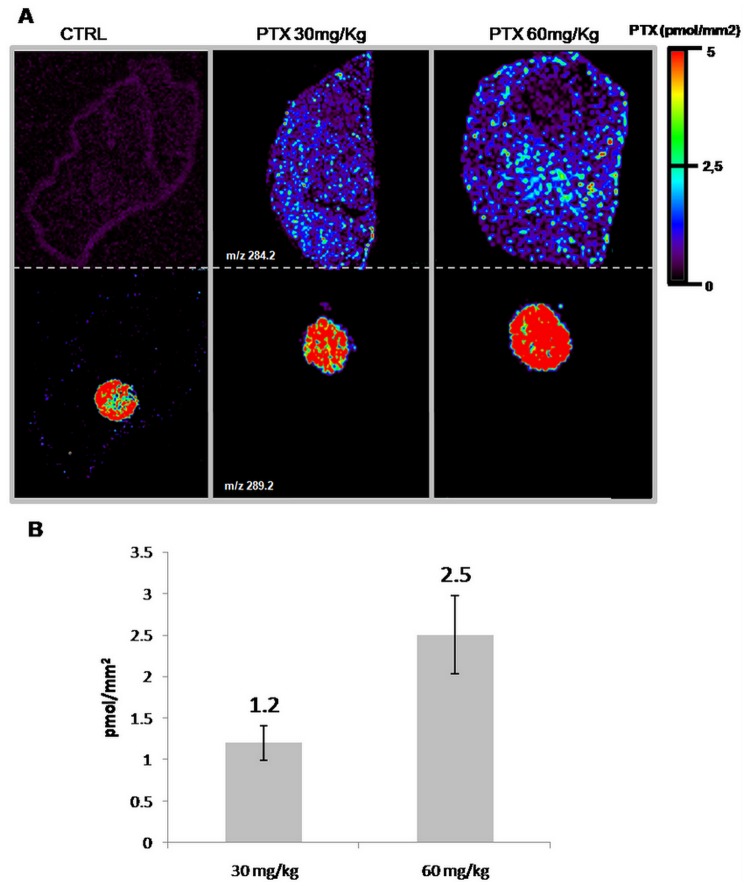
Quantitative analysis by MSI. **A)** MS ion image of PTX (side chain m/z 284.2 upper panel) in liver of a control mouse treated with vehicle and of mice treated with paclitaxel 30 mg/kg or 60 mg/kg, collected after 15 minutes. Lower panel: internal standard (ion m/z 289.2), spotted 5 pmol/mm^2^ on tissue. **B)** PTX abundance estimated from normalized ion signal intensity correlates well with the administered dose. Error bars represent standard deviation (n = 3), p<1% (Student’s t test).

The PTX distribution appears uniform in the liver of treated mice while the signal is absent in control tissue. The images illustrate a clear correlation between the dose of PTX administered and the drug concentration, estimated from normalized ion 284.2 m/z signal intensity using the calibration curve built on the corresponding control tissue (Figure 3).

This conclusion is confirmed by conventional quantitative approach by HPLC-MS/MS: PTX concentration (mean +/− SD; n = 4) in livers from mice treated with 60 mg/kg was 160.8+/−5.3 µg/g, two times higher than in livers from mice treated with 30 mg/kg (90.3+/−10.3 µg/g). Similar results were obtained for kidney sections (data not shown).

### Distribution of PTX Inside Tumor Xenografts

**Figure 4:**
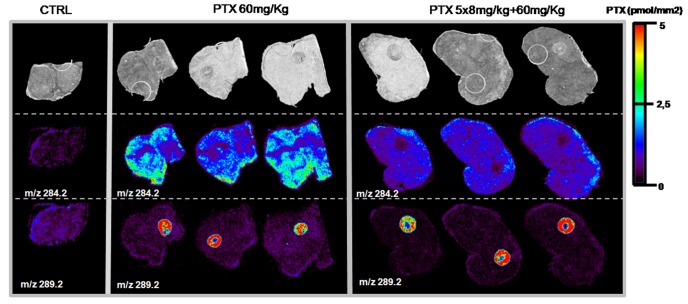
PTX distribution in melanoma xenografts. Treated with vehicle (control, CTRL), 60 mg/kg, pretreated (5×8 mg/kg)+single dose (60 mg/kg) melanoma. Optical image in upper panels, distribution of PTX (ion m/z 284.2) in middle panels and internal standard, spotted 5 pmol/mm^2^ on tissue, in lower panels (ion m/z 289).

Quantitative HPLC-MS/MS determination of PTX in differently treated melanoma xenografts indicate a significantly lower concentration in the tumor homogenate from mice (n = 3) given the single 60 mg/kg dose (20.0+/−3.0 ng/g, mean +/− SD) than in the tumor homogenate from mice that received 5×8 mg/kg plus a 60 mg/kg dose (25.8+/−0.8 ng/g) (t-test p<0.05, test performed with GraphPad Prism, V 6.01). In the second half of tumors used for HPLC-MS/MS, PTX was visualized by MSI in MS and MS/MS mode on three contiguous tissue sections, collected in the central portion of tumors, of control melanoma xenograft treated with vehicle, melanoma xenografts treated with PTX (single 60 mg/kg; n = 3) or pretreated with five low doses of PTX followed by a single high dose (5×8 mg/kg plus final 60 mg/kg, n = 3). PTX distribution inside the melanomas differed with the two schedules (Figure 4). As we mentioned, the images were normalized to 5 pmol/mm^2^ in order to better appreciate distribution differences inside the tumor mass and between different tumors. Mice receiving chronic (5×8 mg/kg) plus a 60 mg/kg dose had more homogeneous drug distribution inside the tumor than after the single 60 mg/kg dose, where the drug distribution is irregular.

**Figure 5:**
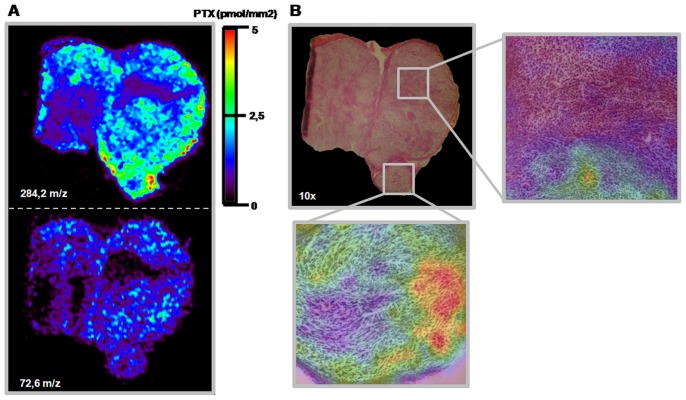
Overlapping MS, MS/MS and HE images of three adjacent slice of melanoma. **A)** MS distribution of peak m/z 284.2 in treated (PTX 60 mg/kg) melanoma reflects MS/MS distribution of the m/z 284.2 to m/z 72,6 transition. **B)** HE stained 10×optical image shows that the cell compartment is uniform. The enlargements show superimposed histological and molecular data. No differences in tissue histology can be seen in the areas where the drug is more concentrated.

The MS/MS image of the transition m/z 284.2 to m/z 72.6 (Figure 5A) can be superimposed on the ion m/z 284.2 signal, confirming the quality and specificity of PTX MSI.

To establish whether the heterogeneous distribution of the drug could be due to non-homogeneous tissue, we performed a comparative histological analysis of an adjacent section of melanoma from mice treated with 60 mg/kg PTX. Figure 5B shows images of PTX distribution, overlapping on optical images of the HE-stained adjacent tumor slice. No histological differences were seen in the zones where the drug is present and where the drug penetration was below our LOD. The tissue section composition was uniform, with no necrotic region, suggesting that the PTX penetration is due to the different treatment schedules.

**Figure 6:**
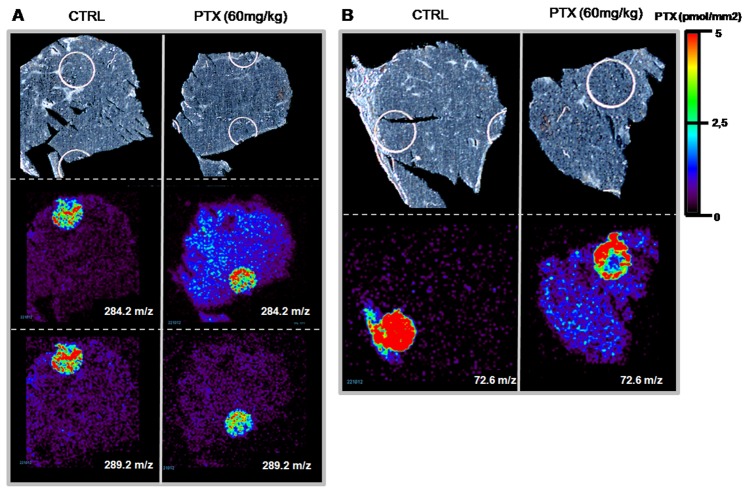
PTX distribution in 1A9 xenografts. Tissue sections of control (CTRL) and PTX-treated (60 mg/kg) ovarian tumors. **A)** Upper panels: optical image; middle panels: distribution of PTX (ion m/z 284), inside tissue and spotted on sections 5 pmol/mm^2^; lower panels: internal standard (ion m/z 289.2), spotted 5 pmol/mm^2^ on tissue. **B)** Optical images in upper panels and MS/MS distribution of ion m/z 72.6 derived from fragmentation of peak m/z 284.2 in lower panels.

To further validate our imaging protocol we analyzed a different tumor type, ovarian carcinoma 1A9, collected 6 h after a single PTX dose of 60 mg/kg (n = 3). Figure 6 shows images of different tumor sections, acquired in MS (m/z 284.2 and m/z 289.2) and in MS/MS mode (m/z 72.6). Similarly to the results with melanomas, PTX can be visualized clearly in both modes only in treated tissue but not in controls; the drug distribution appears homogeneous even without chronic pretreatment. This different distribution could be ascribed to the sampling time (6 h instead of 1 h from dosing) in fact melanoma samples collected 6 h after a single 60 mg/kg dose, show a more regular drug distribution (fig. S5 in File S1). Although the two cancer models are hard to compare, because of their different origins, WM1552/5 melanoma is more highly vascularized than 1A9 ovarian carcinoma [44]. Further studies are need to understand the influence of tumor vasculature on drug distribution.

## Conclusion

We have demonstrated the feasibility of measuring the distribution of PTX in tissues and tumor using the n-PALDI protocol developed for MSI. The technique gives reliable tissue imaging in the low-mass range, feasible using commercially available TiO_2_ nanoparticles as matrix. The uniform nanoparticles deposition over tissues overcomes the variability in signal response due to heterogeneous crystallization of the tissue/matrix complex. It was possible to visualize the different distribution of PTX in tumor and normal tissues, related to the dosage-schedules and pathological features of the tumors. Images are created at a spatial resolution of 75 µm, so microscopy HE-stained tissues images can be superimposed on the MS images and the tissue morphology can be correlated to the drug intake. This protocol can be used to investigate the distribution of anticancer agents in primary tumors and metastases, to ascertain whether resistance is related to inadequate drug penetration in poorly vascularized parts of the tumor, and to develop new methods to enhance anticancer drug tumor uptake and retention, with the aim of increasing their efficacy.

## Supporting Information

File S1 **Figure S1 in file S1.** Orbitrap FT-MS full scan of PTX direct infusion in negative ion mode. Under negative ions, ESI conditions, PTX appears at m/z 898.3275, identified as M+HCOO ]-, with a −0,06 ppm error. **Figure S2 in file S1.** Orbitrap FT-MS2 negative ion scan of m/z 898. Fragment at m/z 284,0926 is identified as the proposed structure with a 2.9 ppm error. **Figure S3 in file S1.** MS3 negative ion scan. The ion at m/z 73 is identified as originating from the m/z 898 ->284 transition. **Figure S4 in file S1.** Mean calibration curve obtained from three experiments performed in different days. The linearity range was found between 1 and 15 pmol/spot with the correlation of estimation of 0.994. The error bar represented the mean ±standard deviation (n = 3). **Figure S5 in file S1.** PTX distribution in melanoma xenografts. Tissue sections of control (CTRL) and PTX-treated (60 mg/kg) melanomas Upper panels: distribution of PTX (ion m/z 284), inside tissue; lower panels: internal standard (ion m/z 289.2), spotted 5 pmol/mm2 on tissue.
(DOCX)
