# Supplementary material for: Determination of Paclitaxel Distribution in Solid Tumors by Nano-Particle Assisted Laser Desorption Ionization Mass Spectrometry Imaging
Source: PLoS One. 2013 Aug 26;8(8):e72532. doi: 10.1371/journal.pone.0072532 (PMC3753243; doi:10.1371/journal.pone.0072532)
Supplement: File S1 — Figure S1 in file S1. Orbitrap FT-MS full scan of PTX direct infusion in negative ion mode. Under negative ions, ESI conditions, PTX appears at m/z 898.3275, identified as M+HCOO ]-, with a −0,06 ppm error. Figure S2 in file S1. Orbitrap FT-MS2 negative ion scan of m/z 898. Fragment at m/z 284,0926 is identified as the proposed structure with a 2.9 ppm error. Figure S3 in file S1. MS3 negative ion scan. The ion at m/z 73 is identified as originating from the m/z 898 ->284 transition. Figure S4 in file S1. Mean calibration curve obtained from three experiments performed in different days. The linearity range was found between 1 and 15 pmol/spot with the correlation of estimation of 0.994. The error bar represented the mean ±standard deviation (n = 3). Figure S5 in file S1. PTX distribution in melanoma xenografts. Tissue sections of control (CTRL) and PTX-treated (60 mg/kg) melanomas Upper panels: distribution of PTX (ion m/z 284), inside tissue; lower panels: internal standard (ion m/z 289.2), spotted 5 pmol/mm2 on tissue. (DOCX) [file pone.0072532.s001.docx]

SUPPORTING INFORMATION


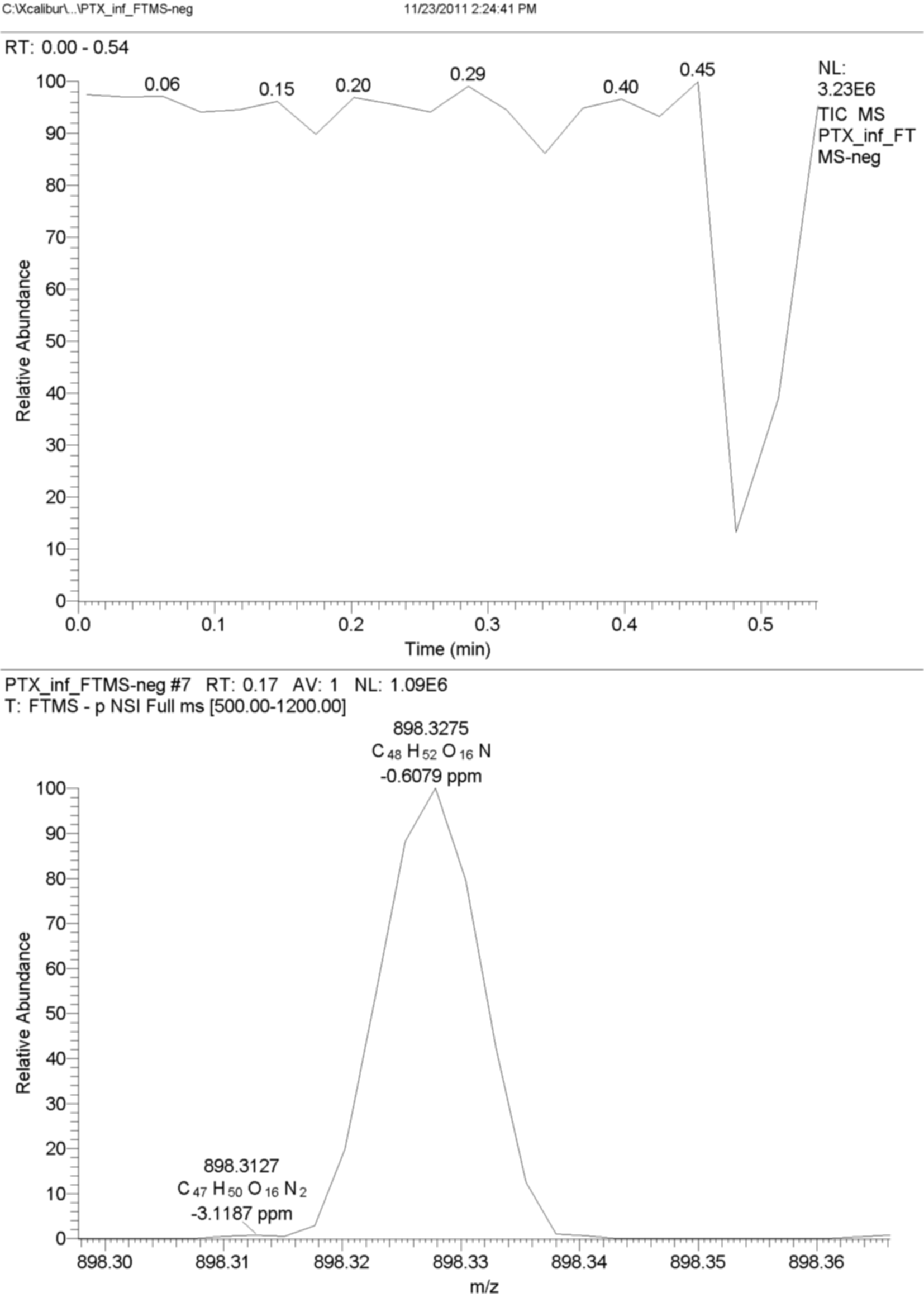


Figure S1. Orbitrap FT-MS full scan of PTX direct infusion in negative ion mode. Under negative ions, ESI conditions, PTX appears at m/z 898.3275, identified as M+HCOO ]- , with a -0,06 ppm error.


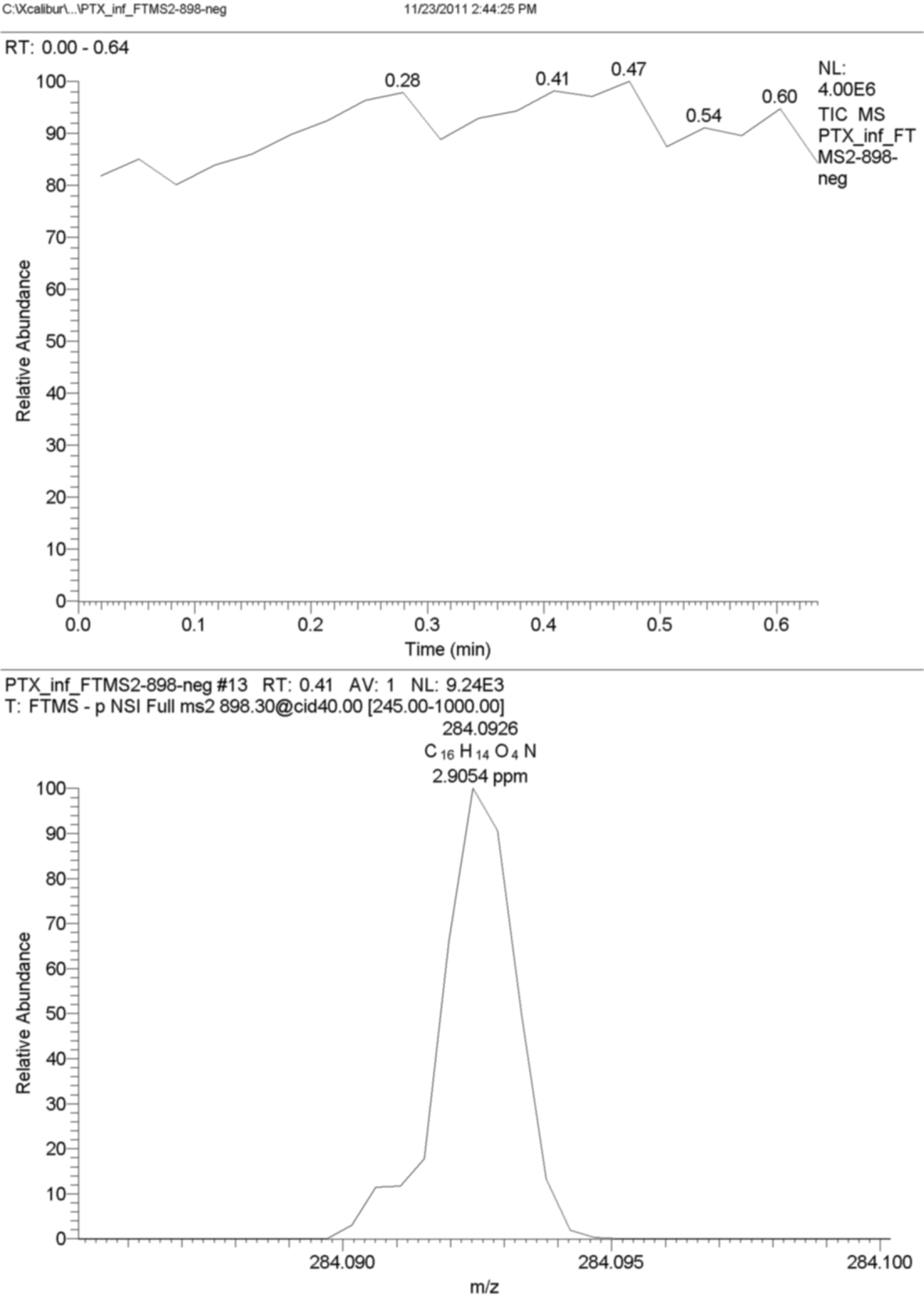


Figure S2. Orbitrap FT-MS2 negative ion scan of m/z 898. Fragment at m/z 284,0926 is identified as the proposed structure with a 2.9 ppm error.





Figure S3. MS3 negative ion scan. The ion at m/z 73 is identified as originating from the m/z 898 -> 284 transition.


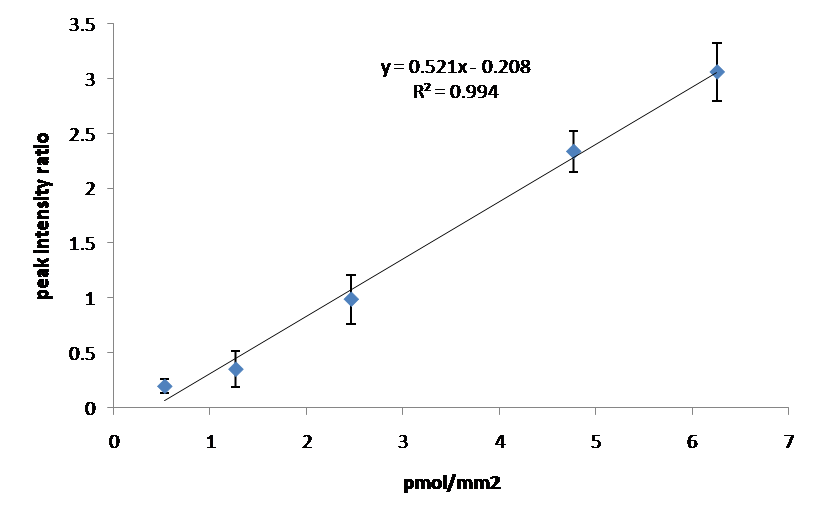


Figure S4. Mean calibration curve obtained from three experiments performed in different days. The linearity range was found between 1 and 15 pmol/spot with the correlation of estimation of 0.994. The error bar represented the mean ±standard deviation (n=3).


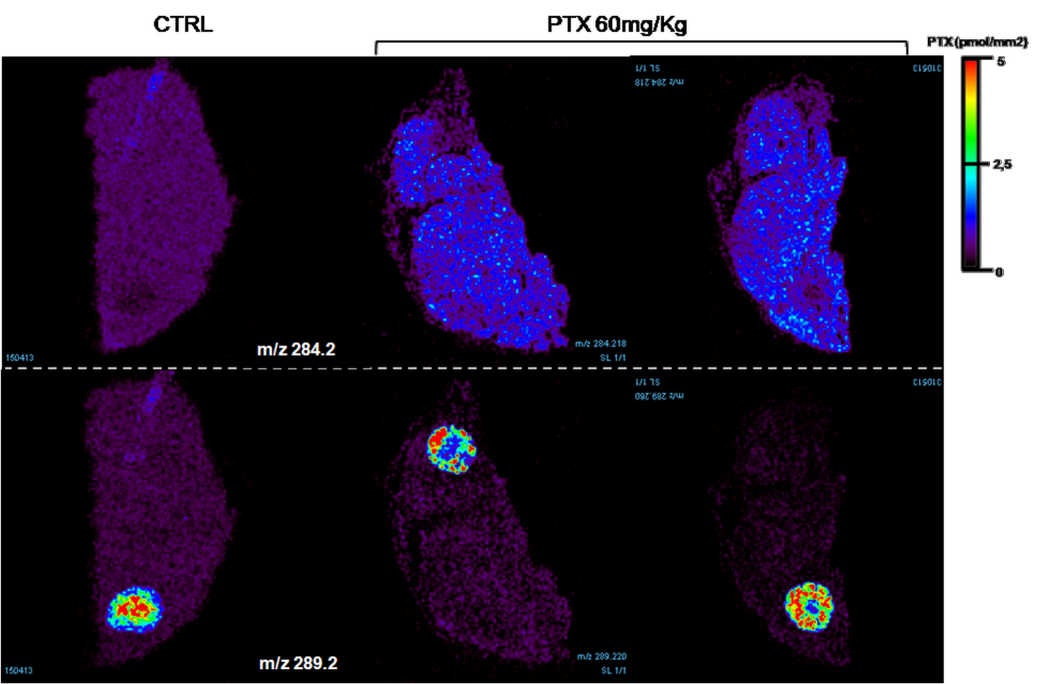


Figure S5. PTX distribution in melanoma xenografts. Tissue sections of control (CTRL) and PTX-treated (60mg/kg) melanomas Upper panels: distribution of PTX (ion m/z 284), inside tissue; lower panels: internal standard (ion m/z 289.2), spotted 5 pmol/mm^2^ on tissue.
